# Fluid removal tolerance during the de-escalation phase: is preload unresponsiveness the best guiding candidate?

**DOI:** 10.1186/s13054-023-04444-3

**Published:** 2023-04-20

**Authors:** Martin Ruste, Rémi Schweizer, Jean-Luc Fellahi, Matthias Jacquet-Lagrèze

**Affiliations:** 1 Service d’anesthésie-Réanimation, Hôpital Louis Pradel, Hospices Civils de Lyon, 59, Boulevard Pinel, 69677 Bron Cedex, France; 2 Faculté de Médecine Lyon Est, Université Claude Bernard Lyon 1, 8, Avenue Rockefeller, 69373 Lyon Cedex 08, France; 3 Laboratoire CarMeN, Inserm UMR 1060, Université Claude Bernard Lyon 1, Lyon, France

**Keywords:** Net ultrafiltration, Fluid removal, Preload responsiveness, Circulatory sufficiency

## Comment

We read with great interest the recent article by Monnet et al. [1] extensively reviewing the various facets of personalized fluid therapy during septic shock. Subsequently, we would like to discuss the management of fluid removal guided by preload responsiveness, specifically regarding the net ultrafiltration (UFnet) set-up in critically-ill patients with continuous renal replacement therapy (CRRT).

A four-phase therapeutic management of shock states was first described by Vincent et al. [2] ten years ago. This management strategy starts with a “salvage” phase that includes lifesaving measures and is followed by an “optimization” phase, which targets the normalization of end-organ perfusion. The latter is performed by means of: i) a preload-responsiveness-guided fluid administration; ii) a mean arterial pressure-guided administration of vasopressors; iii) a cardiac index-guided administration of inotropes. Then, the “stabilization” phase focuses on organ support and the minimization of complications. Finally, the “de-escalation” phase consists in fluid removal if a negative fluid balance is not spontaneously achieved, to counteract the side effects inherent to the initial resuscitation and fluid creep. This strategy, however, appears risky since a discrepancy between the timing and/or intensity of fluid removal rate and the vascular refilling rate may lead to iatrogenic hypovolemia.

Patients with acute kidney injury requiring CRRT display a marked and frequent fluid overload, the peak being observed at day 5 after diagnosis and associated with a worse prognosis [3]. In such patients with inadequate diuresis, the achievement of a negative fluid balance requires a UFnet that allows a real-time fluid removal and which is easily and precisely adjustable. The setting of the UFnet rate is thus particularly illustrative of the complexity of fluid removal management during de-escalation. Several observational studies have shown that a moderate UFnet between 1.01 and 1.75 mL/kg/h is associated with a better prognosis following a J-shaped curve [4]. This suggests that when UFnet is under 1.01 mL/kg/h, the treatment of fluid accumulation is insufficient to counteract the side effects of fluid [5], while a more aggressive strategy (UFnet rate > 1.75 mL/kg/h) may be even more deleterious [6], inducing hypovolemia-related organ damage.

Monnet et al. argue that the absence of preload responsiveness allows the safe initiation of fluid removal, while the apparition of fluid responsiveness during fluid removal is a red flag urging to stop it. Preload responsiveness was reported to predict the occurrence of hypotension during intermittent hemodialysis with a high UFnet rate of 10–15 mL/kg/h [7]. To the best of our knowledge, it is not possible to predict the vascular refilling rate. If it may exceed 10 mL/kg/h in chronic hemodialysis patients [8], the combination of glycocalyx degradation, impaired lymphatic function, and interstitial space architectural modification in critically-ill patients probably lowers considerably this vascular refilling rate [9]. We previously found that passive leg raising does not accurately predict cardiac index decrease nor hypotension following a fluid removal challenge of 500 mL UFnet over one hour in patients undergoing CRRT [10]. We observed the same results with calibrated abdominal compression to test preload responsiveness in children before a diuretics-induced fluid removal of 5 mL/kg over 2 h [11]. In our opinion, preload unresponsiveness better predicts the delay between the initiation of a UFnet at a higher rate rather than that of vascular refilling and the occurrence of hypovolemia. The extrapolation of preload responsiveness to guide fluid removal for resolving interstitial edema in patients without intravascular hypervolemia is questionable. In this situation, we believe that the adequacy between vascular refilling and fluid removal rates is the cornerstone of hemodynamic stability. Moreover, limiting fluid removal to patients with preload unresponsiveness may seriously limit the eligible population.

In this context, what are the other safety criteria that could be used? Arterial hypotension is multifactorial in patients undergoing renal replacement therapy and may be delayed during hypovolemia. Legrand et al. [12] suggested to keep cardiac output constant. Unfortunately, we do not know which cardiac output should be targeted during the de-escalation phase. Indeed, these patients do not experience hypoperfusion and are not necessarily candidates to cardiac output optimization. Variations in cardiac output have no clear prognostic value, and a spontaneous cardiac output decrease can be related to the resolution of a hyperdynamic state in the setting of an overall improvement. It may also not be sensitive enough to detect cutaneous perfusion impairments during fluid removal [13]. As suggested during the optimization phase, targeting circulatory sufficiency (i.e. the normalization of tissue perfusion) [14] seems pertinent. Such a strategy, together with the application of a moderate and continuous UFnet, is feasible and leads to a more negative fluid balance at day 5 than usual practices [15]. Nevertheless, further studies are required to demonstrate the relevance of this approach on patient-centered outcomes.

Fluid removal tolerance is not the only question raised by the concept of de-escalation: the triggers to initiate fluid removal, the targets, and the medication to be used, especially in patients without CRRT, are several of the factors that require elucidation before confirming the relevance of this intellectual concept.

## Data Availability

Not applicable.
